# Effects of Normothermic Hepatic Ischemia–Reperfusion Injury on the Hepatic Disposition of Arachidonic Acid and Its Cytochrome P450-Generated Metabolites in Rats

**DOI:** 10.1007/s11095-026-04108-1

**Published:** 2026-05-23

**Authors:** Vindhya Edpuganti, Reza Mehvar

**Affiliations:** 1 Department of Pharmaceutical Sciences, School of Pharmacy, Texas Tech University Health Sciences Center, Amarillo, TX 79106 USA; 2 Department of Biomedical and Pharmaceutical Sciences, School of Pharmacy, Chapman University, 9401 Jeronimo Road, Irvine, CA 92618 USA

**Keywords:** arachidonic acid, dihydroxyeicosatrienoic acids, epoxyeicosatrienoic acids, hepatic ischemia–reperfusion injury, hydroxyeicosatetraenoic acids

## Abstract

**Purpose:**

Cytochrome P450 (P450) metabolites of arachidonic acid (AA) are potential therapeutic targets in ischemia–reperfusion (IR) injury, yet their disposition and actions in hepatic IR are unknown. Cellular AA and its P450 metabolites are primarily bound to lipid membranes, resulting in negligible free concentrations. This study investigates how hepatic IR injury impacts the disposition of free AA and its major P450 metabolites in rats.

**Methods:**

Rats underwent 60 min of partial (70%) ischemia or sham surgery, followed by 5 min, 3 h, or 24 h of reperfusion. The effects of IR injury on free concentrations of AA and its major P450-generated metabolites in the liver were studied using LC–MS/MS. The activities of phospholipase A_2_ (PLA_2_) enzymes, which release AA and its preformed metabolites from lipid membranes, were also measured in the liver and plasma. Additionally, *in vitro* rates of metabolite formation and the levels of major AA-metabolizing enzymes were assessed in microsomes.

**Results:**

Hepatic IR injury significantly increased free liver concentrations of AA (sevenfold) and its metabolites (1.8–9.1-fold) only in the 5-min group. Similarly, PLA_2_ enzyme activity in the liver and plasma was higher only in this group. Conversely, liver IR injury caused a significant reduction or no change in the *in vitro* rate of AA metabolism to P450-mediated metabolites in the 5-min group.

**Conclusions:**

Liver IR injury rapidly increases liver concentrations of free P450-mediated metabolites of AA, likely due to IR-induced release of preformed metabolites and AA from lipid membranes, rather than an increase in P450 enzymatic activity.

**Supplementary Information:**

The online version contains supplementary material available at https://doi.org/10.1007/s11095-026-04108-1.

## Introduction

Normothermic (warm) hepatic ischemia–reperfusion (IR) injury occurs in a number of clinical situations, such as liver transplantation, partial hepatectomy, and hemorrhagic shock [1, 2]. The injury results from a transient interruption of blood supply to the liver, which may lead to liver failure, systemic inflammatory response, and multiorgan failure [3–5]. The molecular mechanisms of hepatic IR injury are complex, involving multiple liver cells and recruited neutrophils, which trigger a strong inflammatory response. The injury results in the production or release of inflammatory mediators, including reactive oxygen species (ROS), pro-inflammatory cytokines, damage-associated molecular patterns, and adhesion molecules [3, 5, 6]. Despite significant progress in recent years, the complex pathophysiology of the disease remains incompletely understood, and effective therapeutic interventions have yet to emerge.

**Scheme 1:**
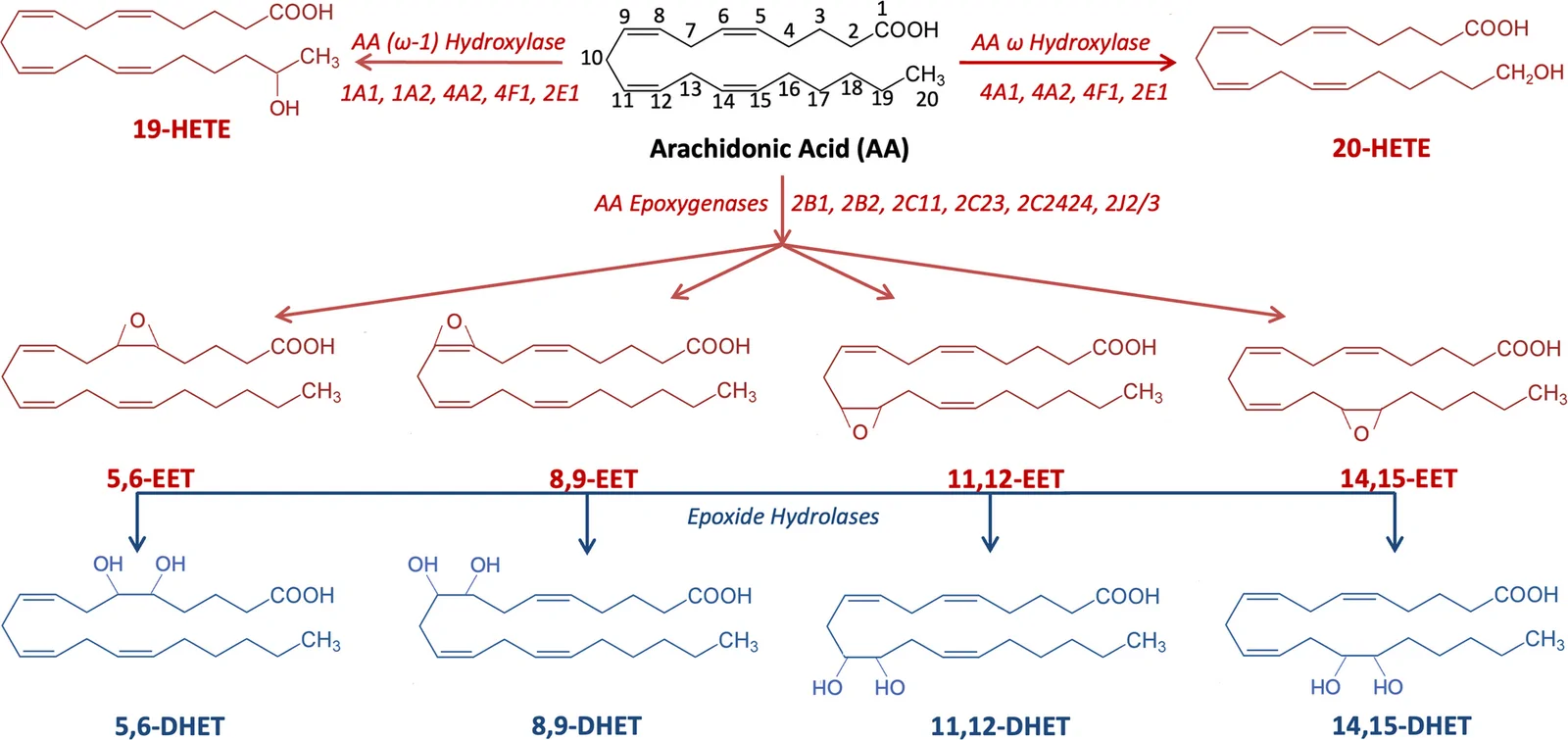
Chemical structures of arachidonic acid (AA) and its ω/(ω−1) hydroxylated (19- and 20-HETE) and epoxygenated (EETs) metabolites formed via P450 enzymes and sequential metabolism of EETs to their respective DHETs via epoxide hydrolases. The cytochrome P450 isoenzymes in the scheme are the major enzymes responsible for each metabolic pathway in rats [25, 43, 63]

Arachidonic acid (AA) is a ω−6 polyunsaturated fatty acid that serves as a structural element in cell membranes and acts as an important precursor to various signaling molecules. AA is metabolized by three pathways, consisting of cyclooxygenases, lipoxygenases, and cytochrome P450 (P450) enzymes, to produce various metabolites with significant pharmacological effects [7–9]. The AA metabolites generated by cyclooxygenases (e.g., prostaglandins and thromboxane A_2_) and lipoxygenases (e.g., leukotrienes and lipoxins) have become major therapeutic targets in many diseases [8, 9]. Additionally, major metabolites of AA generated by the P450 enzymatic pathway include ω/(ω−1) hydroxylation to 19- and 20- hydroxyeicosatetraenoic acid (HETE) and epoxygenation to four regioisomers of epoxyeicosatrienoic acid (EET) (Scheme 1), which are biologically active [7–9]. The epoxygenated metabolites are subsequently converted to less active dihydroeicosatrienoic acid (DHET) metabolites by various forms of the epoxide hydrolase enzymes (Scheme 1) [7–9].

Several studies have shown that the ω/(ω−1) hydroxylated and various regioisomers of the epoxygenated metabolites of AA (Scheme 1) may be therapeutic targets in IR injury to the heart [10–14], brain [15, 16], kidneys [17–19], and lungs [20, 21]. Additionally, recent studies have reported that 12- and 15-HETE metabolites of AA generated by arachidonate 12/15-lipoxygenase (ALOX12/15) play a detrimental role in liver IR injury [22–24]. In addition to numerous other biological activities, such as regulation of intracellular calcium and signaling pathways, both EETs and HETEs are vasoactive compounds [25]. Generally, it is believed that whereas ω/(ω−1)-HETEs (mainly 20-HETE) are vasoconstrictors [26], EETs act as vasodilators [27] in some vascular beds, such as the coronary circulation. However, this is an oversimplification because, whereas 20-HETE is a vasoconstrictor and damaging to the heart and brain during IR injury, it induces medullary vasodilation in the kidneys [28].

In the liver, there is very limited information on the role of 19- and 20-HETE and EETs in the organ’s physiology and pathophysiology [7, 9]. EETs induce vasopressin-mediated glycogenolysis, cause vasoconstriction of the porto-sinusoidal circulation, and contribute to the portal hypertension of cirrhosis [7]. Additionally, EETs stimulate liver regeneration after partial hepatectomy by regulating angiogenesis [9]. The limited data in the liver and the more extensive data in other organs suggest that the P450 metabolites of AA may be therapeutic targets for mitigating IR injury in the liver. However, despite the fact that the liver is the organ with the highest concentration of P450, with demonstrated generation and storage of large quantities of AA metabolites [7], the effects of the P450 metabolites of AA on the liver IR injury are unknown at this time. Therefore, there is a critical need to understand the disposition and actions of P450 metabolites of AA in hepatic IR injury to develop strategies to reduce the injury.

Previous studies have shown that hepatic IR injury affects P450-dependent metabolism by altering the content and function of the P450 enzymes [29–31]. Additionally, IR injury may release AA from its storage sites in lipid membranes [32], potentially altering the formation of its metabolites. Therefore, we hypothesized that hepatic IR injury significantly alters the hepatic concentrations of the P450 metabolites of AA. To test this hypothesis, we investigated the effects of IR injury on the hepatic disposition of the major P450-mediated metabolites of AA, depicted in Scheme 1, in a rat model using a sensitive and specific LC–MS/MS method [33]. Both the *in vivo* liver tissue concentrations and the *in vitro* microsomal rate of production of the P450 metabolites of AA were investigated after the liver IR injury.

## Materials and Methods

### Chemicals

Standards of AA and its metabolites listed below were purchased from Cayman Chemical Company (Ann Arbor, MI): 19-(*R*)-​hydroxy​eicosatetraenoic acid (19-HETE), 20-​hydroxyeicosatetraenoic acid (20-HETE), (±)−5,6-​epoxyeicosatrienoic acid (5,6-EET), (±)−8,9-​epoxyeicosatrienoic acid (8,9-EET), (±)−11,12-​epoxyeicosatrienoic acid (11,12-EET), (±)−14,15-​epoxyeicosatrienoic acid (14,15-EET), (±)−5,​6-​dihydroxy​eicosatrienoic acid (5,6-DHET), (±)−8,​9-​dihydroxy​eicosatrienoic acid (8,9-DHET), (±)−11,​12-​dihydroxyeicosatrienoic acid (11,12-DHET), and (±)−14,​15-​dihydroxyeicosatrienoic acid (14,15-DHET). Deuterated internal standards (20-HETE-d_6_, 8,9-EET-d_11_, 14,15-EET-d_11_,14,15-DHET-d_11_, and AA-d_8_) used for the LC–MS/MS analysis were also obtained from the same company. HPLC grade glacial acetic acid and LC–MS grade acetonitrile were purchased from Fisher Scientific (Fair Lawn, NJ), and LC–MS grade water was purchased from Avantor Performance Materials, Inc. (Center Valley, PA). Cytochrome C from equine heart, β-nicotinamide adenine dinucleotide, reduced disodium salt hydrate or NADH, and isocitric dehydrogenase from porcine heart (Type IV) were obtained from Sigma-Aldrich Co. (St. Louis, MO). β-Nicotinamide adenine dinucleotide phosphate reduced hydrate (NADPH) was purchased from Calzyme Laboratories, Inc. (San Luis Obispo, CA). All other reagents were of analytical grade and purchased from commercial sources.

### Animals

Adult male Sprague Dawley rats (weighing 250–315 g) were purchased from Charles River Laboratories, Inc. (Wilmington, MA, USA) and kept on a 12 h light/dark cycle in the institutional animal facility. They had free access to food and water before the experiments. All animal treatment procedures complied with the guidelines set forth in the Guide for the Care and Use of Laboratory Animals [34] and were approved by our Institutional Animal Care and Use Committee.

### Surgical Procedures and Experimental Groups

A previously described [31, 35] rat model of partial (70%) normothermic hepatic IR was utilized. Briefly, the animals were fasted overnight and then anesthetized via intramuscular injection of ketamine and xylazine (80:10 mg/kg). Ischemia was induced by clamping the portal triad (comprising the portal vein, hepatic artery, and bile duct) associated with the left and median lobes of the liver for 60 min, while maintaining blood supply to the right and caudate lobes to avert portal vein congestion. The rats' body temperature was kept at 37°C during the ischemic interval using a heating plate and an infrared lamp controlled by a TCAT-2AC Controller (Physitemp, Clifton, NJ). At the conclusion of the ischemic period, the clamp was released to facilitate *in vivo* reperfusion of the liver. Before closing the abdomen, 5 mL of sterile saline at 37°C was introduced into the peritoneal cavity to compensate for any volume loss incurred during ischemia. Controls comprised sham-operated (Sham) rats, which underwent an identical procedure with the exception of portal triad clamping. Different groups of rats were euthanized using a carbon dioxide gas chamber at 5 min, 3 h, or 24 h post-reperfusion, and blood samples were collected from the abdominal aorta using a heparinized syringe. Subsequently, the liver was perfused with cold saline to remove any residual blood. The number of animals per group was six, except for the 3- and 24-h Sham groups, which had 7 and 5 animals, respectively. The reperfusion times of 5 min, 3 h, and 24 h were selected to represent the events at the early, intermediate, and late phases of reperfusion [36, 37]. The 5-min group represents events that occur very shortly after ischemia, including the pH paradox, which results from a switch from the acidic pH during ischemia to pH normalization upon reperfusion. The intermediate phase (3 h) reflects oxidative stress and the involvement of cytokines and chemokines. The late phase (24 h) involves inflammatory cells, such as neutrophils, and adhesion molecules [36].

Fresh liver tissues were minced into small pieces; a portion was immediately homogenized in ice-cold methanol for the quantification of AA and its metabolites, and a second portion was used to prepare microsomes, as described below. The remaining liver tissue was snap-frozen in liquid nitrogen and stored at −80°C.

### Measurement of Liver Injury Markers

Alanine aminotransferase (ALT) and aspartate aminotransferase (AST) levels in plasma were measured using kits from Teco Diagnostics (Anaheim, CA). The ALT assay is linear up to 150 IU/L, whereas the AST assay is linear up to 500 IU/L. The plasma samples from the IR rats were diluted tenfold, while Sham samples were used undiluted or diluted fivefold with distilled water to fit within the assays’ linear ranges.

### Estimation of Phospholipase A_2_ Activity in Plasma and Liver Tissue

Three types of phospholipase A_2_ (PLA_2_) activities were measured: secretory PLA_2_ (sPLA_2_) in plasma, total PLA_2_, which is the sum of sPLA_2,_ calcium-dependent cytosolic PLA_2_ (cPLA_2_), and calcium-independent PLA_2_ (iPLA_2_) in the liver, and liver cPLA_2_ plus iPLA_2_. The activities of various PLA_2_ enzymes were measured using commercial assay kits (Cayman Chemical Company, Ann Arbor, MI), following the manufacturer’s instructions.

The activity of plasma sPLA_2_ was determined using the sPLA_2_ Assay Kit, which uses 1,2-dithio analog of diheptanoyl phosphatidylcholine as a substrate. After hydrolysis of the substrate at the *sn*-2 position, the free thiols of the lysophospholipid are then detected using DTNB [(5, 5^‘^-dithio-bis-(2-nitrobenzoic acid)].

The cPLA_2_ Assay Kit was used to quantify total PLA_2_ activity in the liver. Similar to the sPLA_2_ kit, the assay detects free thiol using DTNB, but the substrate is arachidonoyl thio-phosphatidylcholine. The cPLA_2_ Assay Kit was also used for the quantitation of the liver cPLA_2_ plus iPLA_2_ activity after first treating the liver homogenate with thioetheramide-phosphatidylcholine to inhibit the sPLA_2_ activity.

### Preparation of Liver Microsomes and Measurement of Microsomal Cytochrome P450 Reductase, Cytochrome *b*_*5*_, and Epoxide Hydrolase

Microsomes were prepared using established ultracentrifugation techniques [38]. The homogenization buffer used was sucrose-mannitol buffer (70 mM sucrose, 220 mM mannitol, 20 mM HEPES, 2 mM EDTA, 0.5 mg/mL BSA, pH 7.4), and the storage buffer contained 0.05 M phosphate buffer pH 7.4, 20% v/v glycerol, 0.01 mM EDTA solution, 0.01 mM PMSF solution in IPA, and 1.5% w/v DTT. Microsomal samples were stored at −80°C until analysis.

Microsomal cytochrome P450 reductase (CPR) activity was determined using established procedures [39] with minor modifications. The 1-mL reaction volume contained microsomal proteins (15 µg), cytochrome C (50 µM), and NADPH (200 µM) in a 0.3 M potassium phosphate buffer (pH 7.7). The mixture was first preincubated at 30°C in the absence of NADPH for 3 min. Subsequently, NADPH was added, and the absorbance was recorded at 550 nm for 4 min (30°C). The CPR activity was then estimated using the slope of the linear portion of the plot and an extinction coefficient of 21 mM^−1^.cm^−1^.

The cytochrome *b*_*5*_ content of the liver was measured by spectrophotometry using the method of Omura and Sato [40]. The assay is based on the absorbance difference between NADH-reduced microsomal preparation, measured at 424 nm, and oxidized microsomes, measured at 409 nm in the absence of NADH, and an extinction coefficient of 185 mM^−1^. cm^−1^ for cytochrome *b*_*5*_ [38].

Microsomal epoxide hydrolase (mEH) activity was measured according to the procedure described by Zeldin *et al*. [41]. The microsomes were diluted to 1 mg/mL in a buffer containing 150 mM KCl, 10 mM MgCl_2_, and 50 mM Tris–Cl buffer (pH 7.5) and pre-incubated for 5 min at 37°C. Next, 100 µL of the microsomal suspension was added to a silanized glass tube containing evaporated 14,15-EET (6.25 µM). After vortex-mixing for 15 s, the tube was immersed in a water bath (30°C) and incubated for 10 min. The reaction was stopped by the addition of 900 µL of ice-cold acetonitrile. The samples were processed for measurement of the formed 14,15-DHET, as described in detail below.

### Protein Expression of CYP4A, CYP2C23, and CYP2J2 in Liver Microsomes

The protein contents of CYP4A, 2J2, and 2C23 in liver microsomes were evaluated by Western blot analysis. After boiling the microsomal samples diluted in Laemmli buffer (Bio-Rad Laboratories, Hercules, CA), containing β-mercaptoethanol, equal amounts of microsomal proteins (20 µg per lane) were resolved by 10% SDS–polyacrylamide gel electrophoresis. Subsequently, the gel was transferred to a PVDF membrane and incubated with the respective primary antibodies. Antibodies for CYP4A (ABR Affinity BioReagents, Golden, CO), CYP2C23 (Abcam, Cambridge, MA), and CYP2J2 (Santa Cruz Biotechnology, Inc., Dallas, Texas) were all rabbit polyclonal antibodies reactive with rat antigens. An enhanced chemiluminescent Western blotting system (Pierce Biotechnology, Inc., Rockford, IL) was used to visualize immunoreactive bands. The density of each CYP enzyme band was determined by developing the membrane with a VersaDoc MP 4000® (Bio-Rad Laboratories, Hercules, CA) and quantifying with UN-SCAN-IT Gel Analysis Software (Silk Scientific, Inc., Orem, UT). We used calreticulin (CRT), an endoplasmic reticulum marker, as the housekeeping protein for density normalization. After visualization of the P450 bands, the blots were stripped and re-probed with a goat polyclonal anti-calreticulin antibody against rat (Santa Cruz Biotechnology, Inc., Dallas, Texas). The band density of CRT was then quantified as described above and used to calculate the relative density of the CYP bands.

### *In Vitro *Metabolism of Arachidonic Acid by Rat Microsomes

The rate of production of P450-mediated epoxygenated (EETs) and ω/(ω−1) hydroxylated (HETEs) metabolites of AA and the epoxide hydrolase-mediated metabolites of EETs (DHETs) were determined in microsomes obtained from the Sham and IR animals. A method previously reported [42] was used with some modifications as follows. To a silanized glass tube was added 250 µL of an ethanolic solution of sodium arachidonate (31.25 or 250 µM), which was subsequently evaporated under nitrogen gas. The residue was then dissolved in a 1-mL incubation buffer by vortex mixing, resulting in AA concentrations of 7.81 or 62.5 µM. These concentrations represent values several-fold below (7.81 µM) or around or above (62.5 µM) *K*_*m*_ for the formation rates of EETs and 19- or 20-HETE in rat liver microsomes [43]. The incubation buffer consisted of 50 mM Tris-chloride (pH 7.5), 150 mM KCl, 10 mM MgCl_2_, 8 mM sodium isocitrate, 0.5 IU/mL isocitrate dehydrogenase, and 0.5 mg/mL microsomal protein. The preparation was pre-incubated at 30°C for 3 min in an open water bath with constant stirring, and the reaction was started by the addition and mixing of NADPH (1 mM). The reaction was incubated for 5 min, then stopped by adding 100 µL to 900 µL of ice-cold acetonitrile, after which AA and its metabolites were analyzed as described below.

### Analysis of Arachidonic Acid and Its Metabolites in Liver Tissues or Microsomal Incubations

The concentrations of AA and its P450-mediated metabolites in liver tissues and in microsomal incubations were quantified using a previously reported specific and sensitive UHPLC-MS/MS method [33]. Briefly, the UHPLC-MS/MS instrument consisted of an AB SCIEX QTRAP® 5500 mass spectrometer (Foster City, CA, USA) attached to a Nexera UHPLC system from Shimadzu Corporation (Columbia, MD). The analytes were separated on a Kinetex C_18_ column (5 cm × 2.10 mm I.D., 1.7 µm particle size, 100 Å pore size), preceded by a SecurityGuard ULTRA guard column (Phenomenex; Torrance, CA, USA), which was kept at 40°C.

As described in detail before [33], fresh liver tissue (100 mg) was homogenized in 950 µL of ice-cold methanol containing 0.01 M BHT, 50 µL of a composite internal standard solution was added, and the samples were vortex-mixed and centrifuged at 0°C. The supernatant was stored at −80°C before analysis. On the day of analysis, the methanolic supernatants of the liver homogenates were dried under nitrogen gas, reconstituted in 100 µL of ethanol, and subjected to another centrifugation at 0°C. The final supernatant was divided into two parts for separate analysis of AA and the remaining analytes. For AA analysis, the supernatant was diluted 4000-fold with ethanol before injection into the UHPLC-MS/MS. For metabolite analysis, the supernatant was used directly without dilution. The lower limit of quantitation of the assay was 0.94 ng/g for 19- and 20-HETE, 0.38 ng/g for 5,6-, 11,12-, and 14,15-EET and 8,9-DHET, 0.75 ng/g for 8,9-EET, 0.19 ng/g for 5,6-DHET, 0.48 ng/g for 11,12-DHET, and 1.88 ng/g for 14,15-DHET [33]. For presentation purposes, concentrations below the limit of quantitation (BLQ) are reported as half of the lower limit of quantitation.

For the analysis of analytes in the microsomes, after stopping the microsomal incubation medium (100 µL) with 900 µL of ice-cold acetonitrile, the contents were vortex-mixed, centrifuged at 19,500 g for 10 min at 0°C, and the supernatant was separated*.* Subsequently, 5 µL of a mixture of various internal standards was added to 200 µL of the supernatant, and 3 µL was injected into UHPLC-MS/MS. The calibration standards were prepared by spiking the incubation buffer containing denatured microsomes with stock solutions of the standards [33].

### Statistical Analysis

We analyzed the data using Prism software (GraphPad Software, La Jolla, CA) with two-way ANOVA and the Bonferroni multiple comparisons test for post-hoc analysis. The two factors were the surgery (Sham or IR) and the reperfusion time (5 min, 3 h, and 24 h). The post-hoc analysis compared the means of Sham and IR groups within each reperfusion time and the means of each surgical group (Sham or IR) across different reperfusion times. We also used multiple linear regression to determine the relationships between liver concentrations of each P450 metabolite (dependent variable) and AA (independent variable), with surgical group as a categorical variable. The regression equation was $Metabolite\, Concentration= {\beta}_{0}+{\beta}_{1}\times Group+{\beta}_{2}\times AA \,Concentration$, where ${\beta}_{0}$, ${\beta}_{1}$, and ${\beta}_{2}$ are the intercept, the intercept shift due to the category (Sham or IR), and the slope of the regression between metabolite and AA concentration, respectively. In all cases, a *p* value of < 0.05 was considered significant. The data are presented as mean ± SE.

## Results

### Plasma Markers of Liver IR Injury

**Fig. 1:**
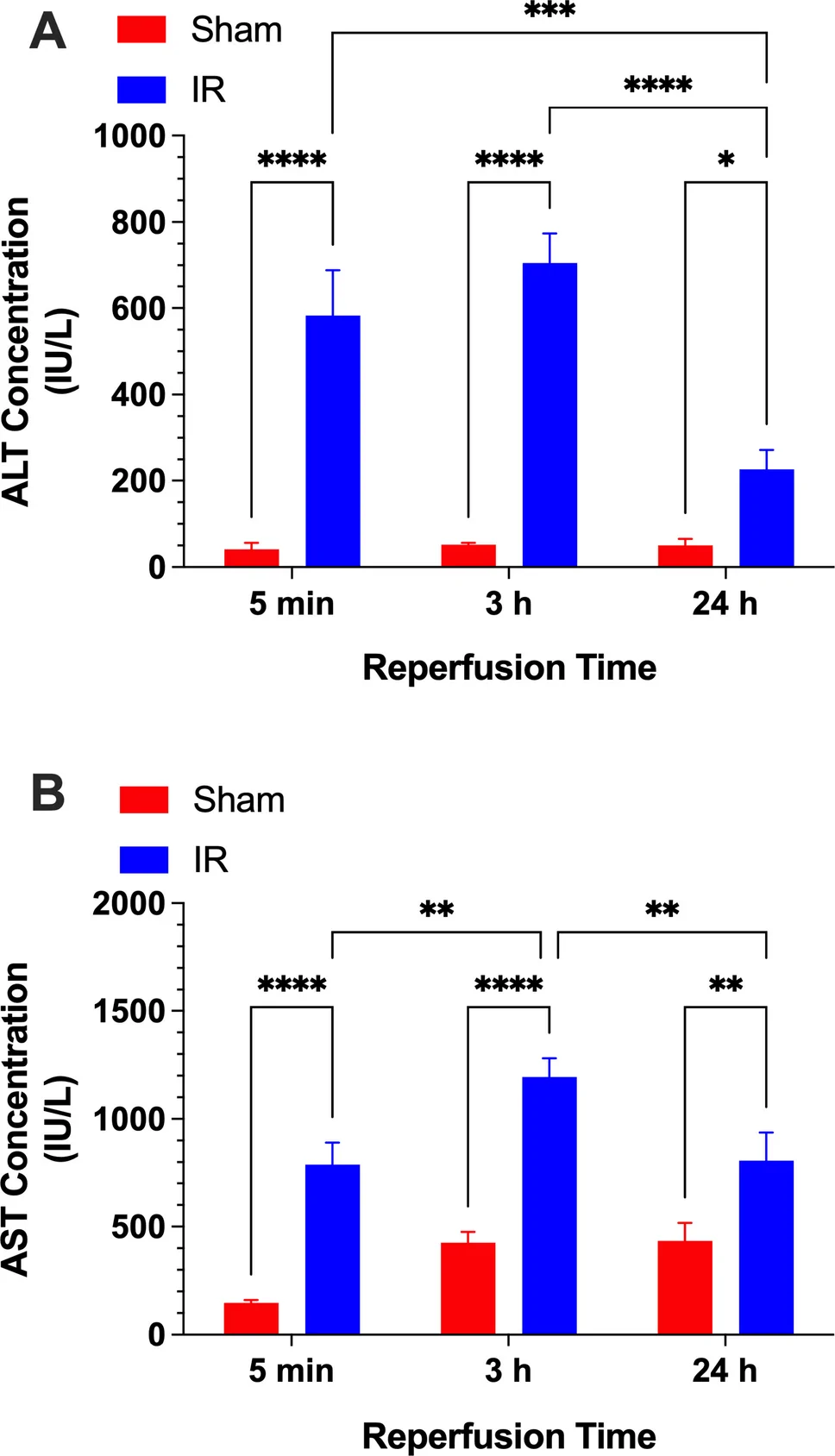
The plasma concentration–time courses of ALT (**A**) and AST (**B**) in rats subjected to partial hepatic ischemia (IR) or sham operation (Sham) and 5 min, 3 h, and 24 h of reperfusion. Columns and bars represent mean and SE values, respectively. The number of animals per group was 6, except for the 3- and 24-h Sham groups, which had 7 and 5 animals, respectively. * *p* < 0.05, ** *p* < 0.01, *** *p* < 0.001, **** *p* < 0.0001: Significant difference between Sham and IR at each time point or between different reperfusion times for Sham or IR, based on two-way ANOVA, followed by Bonferroni’s post-hoc analysis.

Figure 1 depicts the plasma concentrations of transaminases ALT (Fig. 1A) and AST (Fig. 1B) in the Sham and IR animals after 5 min, 3 h, and 24 h of reperfusion. Liver IR resulted in significant increases in the plasma levels of both liver injury markers at all the reperfusion times. The ALT levels in the IR animals were 14.0- (*p* < 0.0001), 13.5-(*p* < 0.0001), and 4.5-(*p* < 0.05) fold higher than the Sham animals after 5 min, 3 h, and 24 h of reperfusion, respectively (Fig. 1A). Similarly, the AST levels in the IR animals after 5 min, 3 h, and 24 h of reperfusion were, respectively, 5.3-(*p* < 0.0001), 2.8-(*p* < 0.0001), and 1.9-(*p* < 0.01) fold higher than those in their Sham counterparts (Fig. 1B). In addition to the significant effects of IR, ANOVA revealed that the ALT and AST levels were also significantly (*p* < 0.001) affected by the reperfusion time, including significant decreases in the 24-h IR animals relative to the values in the 3-h IR rats (Fig. 1).

### Effects of Hepatic IR Injury on the Free (Unesterified) Concentrations of AA in the Liver and the Liver and Plasma Activities of PLA_2_

**Fig. 2:**
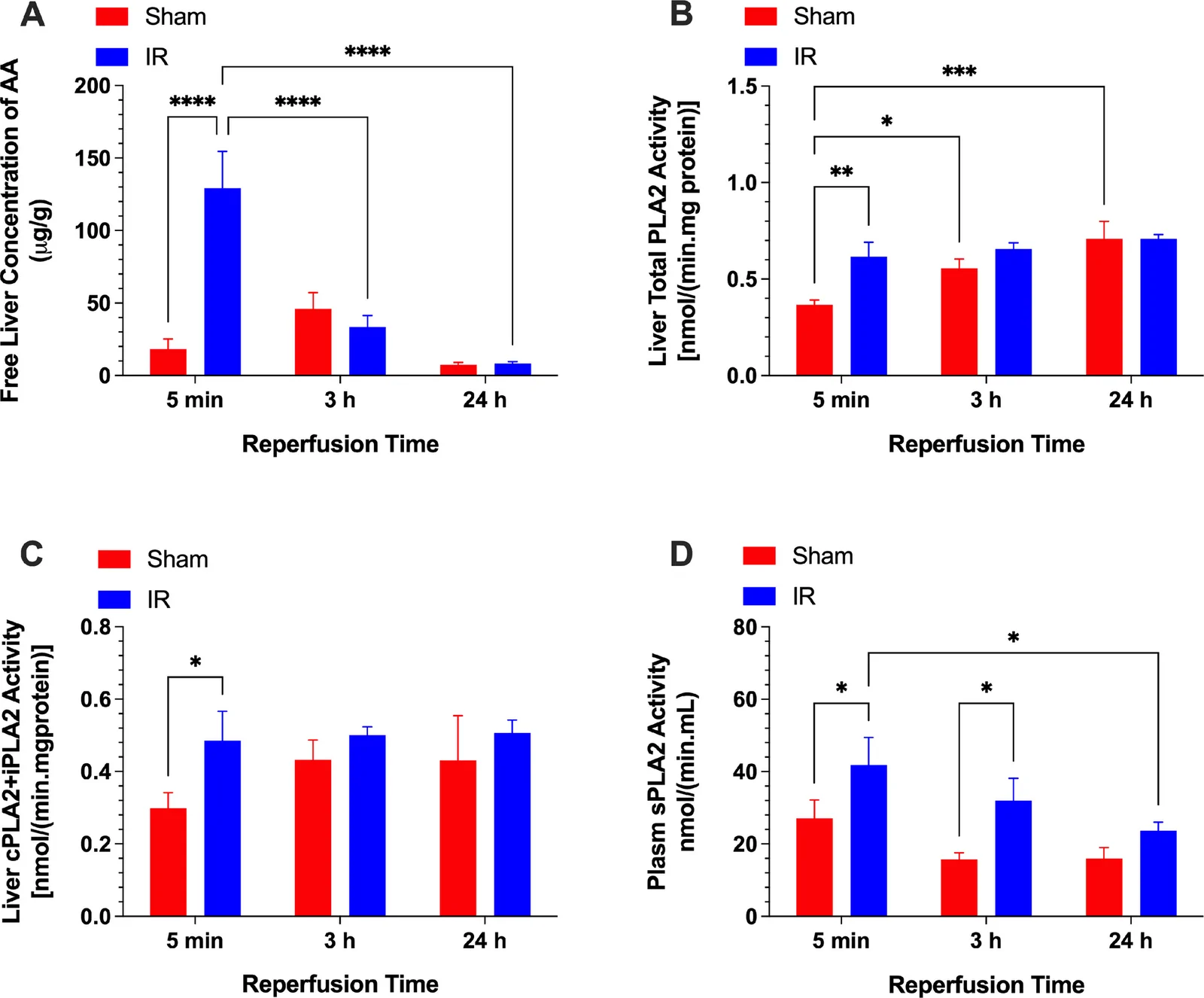
The liver concentrations of the free AA (**A**), along with the activities of total PLA_2_ (**B**) and cPLA_2_ plus iPLA_2_ (**C**) in the liver and sPLA_2_ in the plasma (**D**) in rats subjected to partial hepatic ischemia (IR) or sham operation (Sham) and 5 min, 3 h, and 24 h of reperfusion. Columns and bars represent mean and SE values, respectively. The number of animals per group was 6, except for the 3- and 24-h Sham groups, which had 7 and 5 animals, respectively.* *p* < 0.05, ** *p* < 0.01, *** *p* < 0.001, **** *p* < 0.0001: Significant difference between Sham and IR at each time point or between different reperfusion times for Sham or IR, based on two-way ANOVA, followed by Bonferroni’s post-hoc analysis.

Figure 2 illustrates the liver concentrations of the unbound AA (Fig. 2A), along with the activities of total PLA_2_ (Fig. 2B) and cPLA_2_ plus iPLA_2_ (Fig. 2C) in the liver and sPLA_2_ in the plasma (Fig. 2D). Five minutes after the reperfusion, the free concentrations of AA in the liver of Sham and IR rats were 18.2 ± 7.1 and 129 ± 25 µg/g, respectively, a sevenfold increase (*p* < 0.0001) because of the IR injury (Fig. 2A). However, the AA concentrations in the liver of IR rats after 3 or 24 h of reperfusion were significantly lower than those in the 5 min group, resulting in no significant differences between the Sham and IR rats at 3 or 24 h of reperfusion (Fig. 2A).

The significantly (*p* < 0.0001) higher level of AA in the livers of the 5-min reperfusion group (Fig. 2A) was associated with significant increases in the activities of total PLA_2_ (*p* < 0.01) (Fig. 2B) and cPLA_2_ plus iPLA_2_ (*p* < 0.05) (Fig. 2C) in the liver tissue of these animals, without any significant changes in the activities of these PLA_2_s in the other reperfusion time points. For plasma sPLA_2_ activity, IR caused significant (*p* < 0.05) increases in both the 5-min and 3-h groups (Fig. 2D).

### Effects of Hepatic IR Injury on the Free (Unesterified) Concentrations of ω/(ω −1) HETEs, EETs, and DHETs in the Liver

**Fig. 3:**
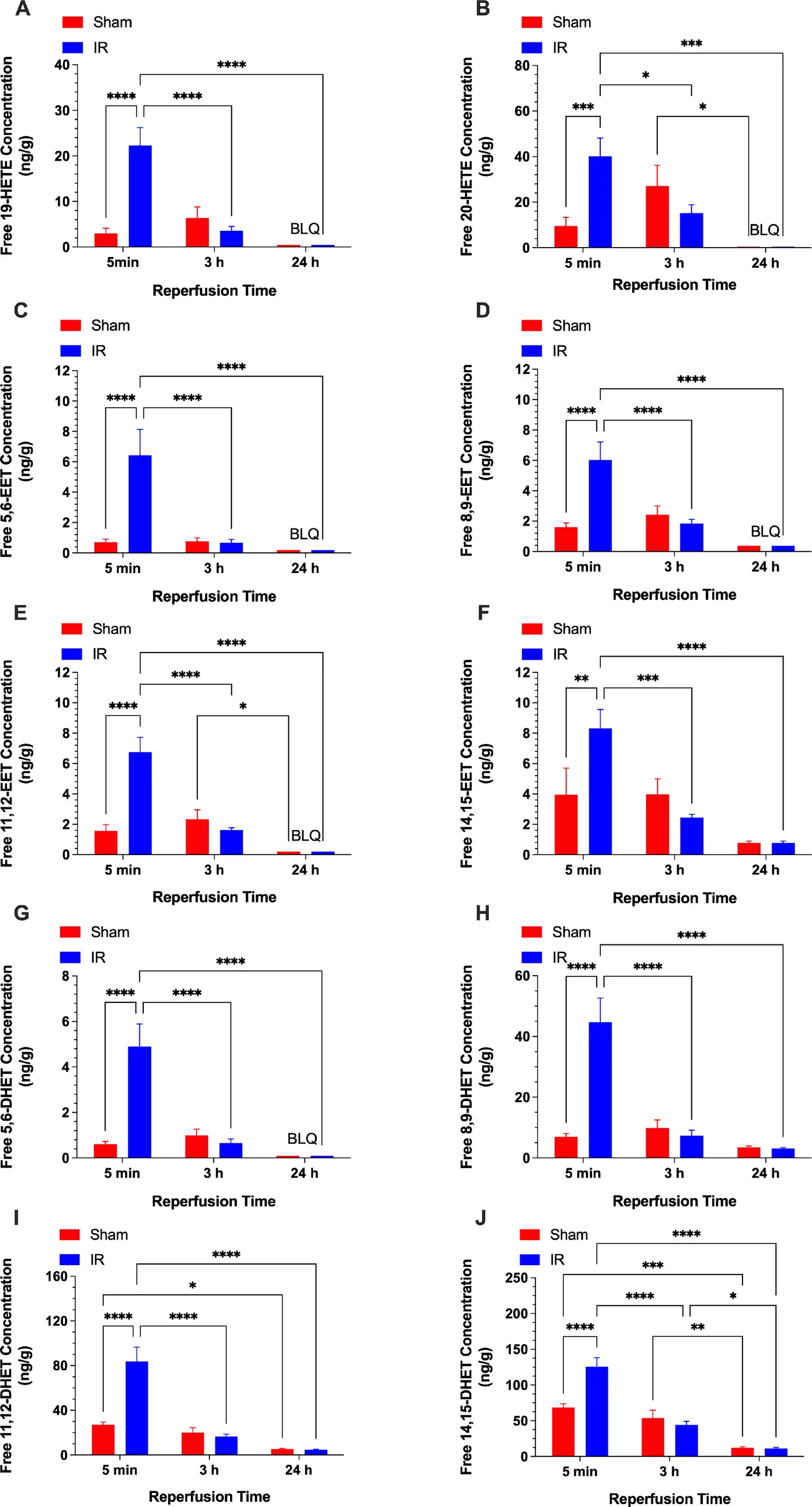
The free concentrations of ω/(ω−1) HETEs (**A** and **B**), EETs (**C**-**F**), and DHETs (**G**-**J**) in the liver tissue of rats subjected to partial hepatic ischemia (IR) or sham operation (Sham) and 5 min, 3 h, and 24 h of reperfusion. Columns and bars represent mean and SE values, respectively. The number of animals per group was 6, except for the 3- and 24-h Sham groups, which had 7 and 5 animals, respectively.* *p* < 0.05, ** *p* < 0.01, *** *p* < 0.001, **** *p* < 0.0001: Significant difference between Sham and IR at each time point or between different reperfusion times for Sham or IR, based on two-way ANOVA, followed by Bonferroni’s post-hoc analysis. BLQ, below the limit of quantitation of the assay.

The free concentrations of ω/(ω−1) HETEs, EETs, and DHETs in the liver tissue of Sham and IR rats after different reperfusion times are presented in Fig. 3. Similar to the pattern for the liver concentrations of free AA (Fig. 2), the free concentrations of all the studied metabolites in the IR animals were significantly higher than those in their Sham controls only in the 5-min group (Fig. 3); there were no significant differences between Sham and IR rats in their liver concentrations of the metabolites at 3 or 24 h reperfusion time (Fig. 3).

The free liver concentrations of 19- (Fig. 3A) and 20-(Fig. 3B) HETE in the 5-min IR rats were 7.5- (*p* < 0.0001) and 4.2- (*p* < 0.001) fold higher than their respective Sham counterparts. Other observations for the 19- and 20-HETE metabolites included an overall higher free liver concentration for 20-HETE, relative to 19-HETE, and below limit of quantitation (BLQ) levels of both metabolites in the 24-h groups (Fig. 3A and B).

Averages of the free liver concentrations of epoxygenated metabolites of AA (Fig. 3C-F) were generally very low (< 10 ng/g) for all four regioisomers of the metabolites and both surgical groups. However, compared with their Sham counterparts, the 5-min IR group had 9.1- (*p* < 0.0001), 3.8- (*p* < 0.0001), 4.3- (*p* < 0.0001), and 2.1- (*p* < 0.01) fold higher concentrations of 5,6-EET (Fig. 3C), 8,9-EET (Fig. 3D), 11,12-EET (Fig. 3E), and 14,15-EET (Fig. 3F), respectively. Similar to the HETE data (Fig. 3A and B), there was a trend toward lower free EET concentrations in the 24-h group, with most being BLQ, compared with earlier reperfusion times (Fig. 3C-F).

The free liver concentrations of DHETs, generated from the hydroxylation of EETs by the enzyme epoxide hydrolase, are presented in Fig. 3G-J. Aside from 5,6-DHET (Fig. 3G), the free liver concentrations of the DHETs were much higher than the concentrations of their precursor EETs (Fig. 3C-F). Compared with their Sham counterparts, the 5-min IR group had 8.2-, 6.5-, 3.1-, and 1.8- fold higher (*p* < 0.0001) concentrations of 5,6-DHET (Fig. 3G), 8,9-DHET (Fig. 3H), 11,12-DHET (Fig. 3I), and 14,15-DHET (Fig. 3J), respectively. Additionally, there was a trend toward lower DHET concentrations with increasing reperfusion time (Fig. 3G-J).

**Fig. 4:**
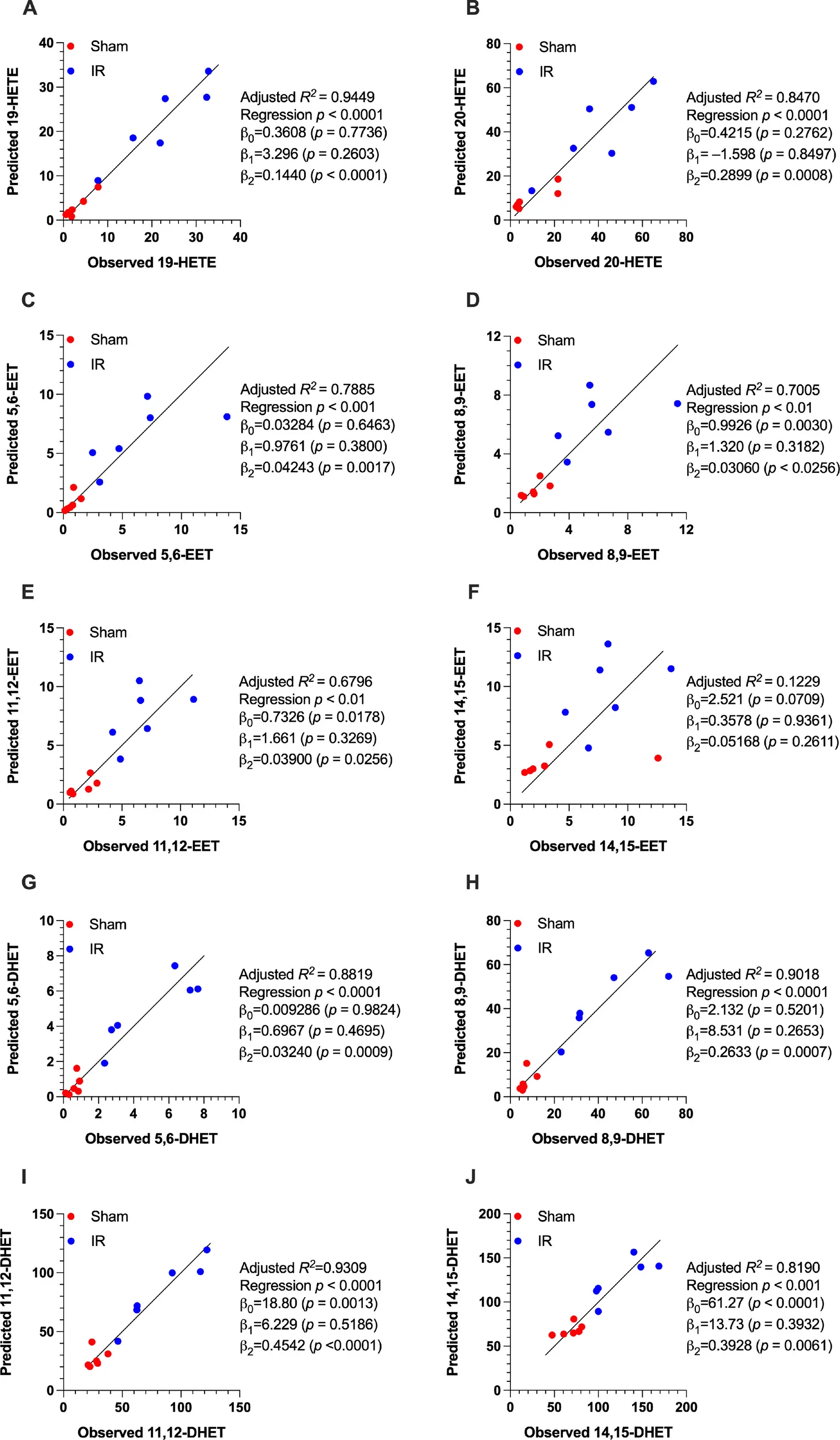
Plots of multiple regression model-predicted liver concentrations of metabolites versus their observed concentrations, along with the statistical data for the multiple regression analysis of the free concentrations of the measured metabolites against the free concentrations of AA, with Sham and IR groups as a categorical variable. The data are presented for ω/(ω−1) HETEs (**A** and **B**), EETs (**C**-**F**), and DHETs (**G**-**J**) for rats subjected to partial hepatic ischemia (IR) or sham operation (Sham) and 5 min of reperfusion. The symbols and the lines represent the data for individual rats and the line of unity, respectively. Plots and associated data are based on the equation $Metabolite\, Concentration= {\beta}_{0}+{\beta}_{1}\times Group+{\beta}_{2}\times AA\ Concentration$.

The results of the multiple linear regression analysis of the free concentrations of the measured metabolites against the free concentrations of AA, with Sham and IR groups as a categorical variable, are depicted in Fig. 4 for the 5-min group. The slopes of the regression of metabolite concentrations against AA concentrations (${\beta}_{2}$) were significant for all the measured metabolites except for 14,15-EET, with no significant effect of surgery (${\beta}_{1}$) for any metabolite (Fig. 4). The adjusted *R*^*2*^ values for the regression model were the highest for HETEs (*R*^*2*^: 0.84–0.94) and DHETs (*R*^*2*^: 0.82–0.93) and lower for EETs (*R*^*2*^: 0.12–0.79) (Fig. 4).

### Effects of Hepatic IR Injury on the *In Vitro* Metabolism of AA to ω/(ω −1) HETEs, EETs, and DHETs in the Liver Microsomes

**Fig. 5:**
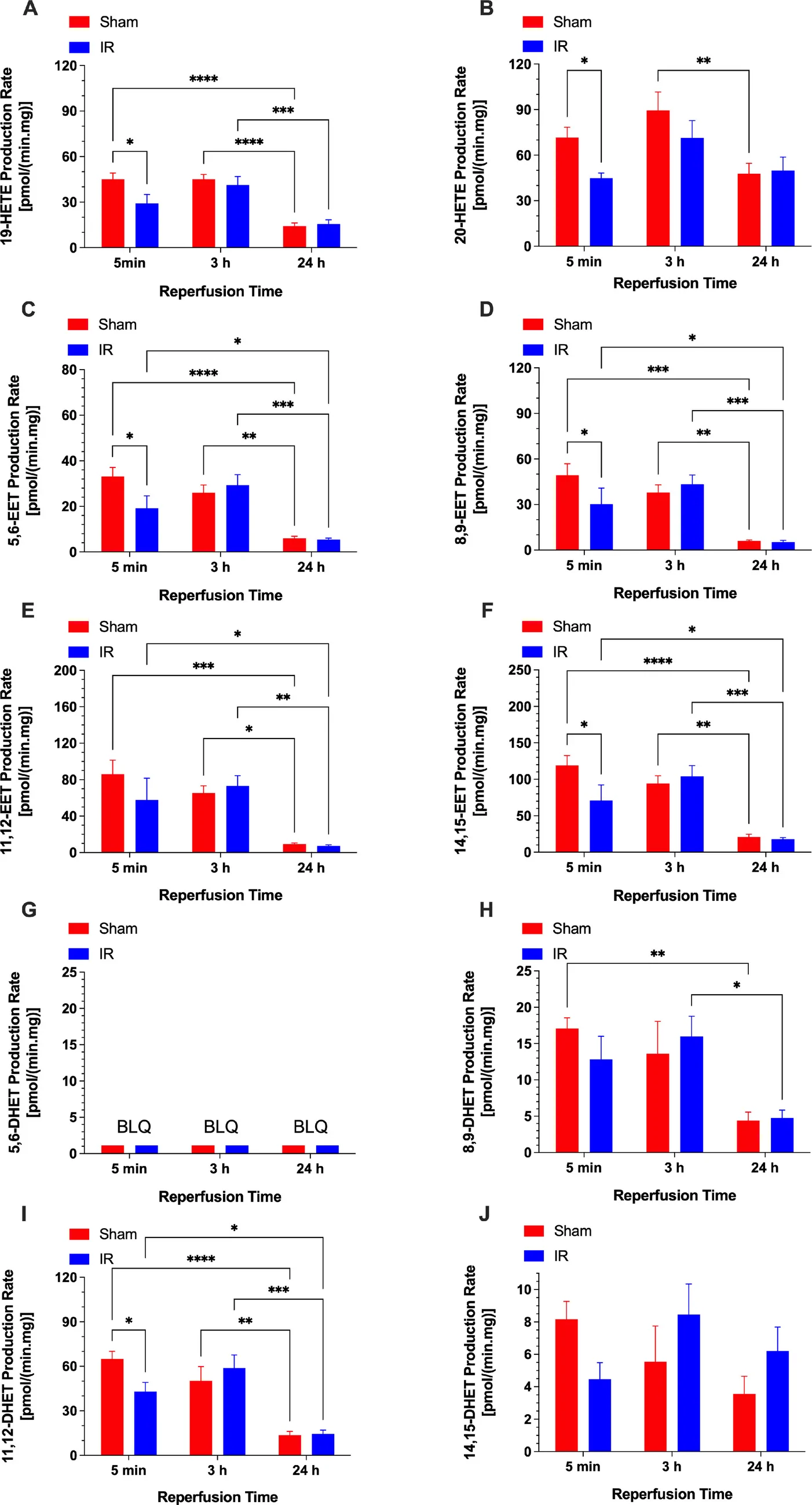
The *in vitro* rate of metabolism of AA (7.81 µM) to HETEs (**A** and **B**) and EETs (**C**−**F**) and sequential metabolism of EETs to their dihydroxylated (DHETs) metabolites (**G**–**J**) in the liver microsomes of rats subjected to partial hepatic ischemia (IR) or sham operation (Sham) and 5 min, 3 h, and 24 h of reperfusion. Columns and bars represent mean and SE values, respectively. The number of animals per group was 6, except for the 3- and 24-h Sham groups, which had 7 and 5 animals, respectively.* *p* < 0.05, ** *p* < 0.01, *** *p* < 0.001, **** *p* < 0.0001: Significant difference between Sham and IR at each time point or between different reperfusion times for Sham or IR, based on two-way ANOVA, followed by Bonferroni’s post-hoc analysis. BLQ, below the limit of quantitation of the assay.

Figure 5 demonstrates the *in vitro* rate of metabolism of AA to HETEs (Fig. 5A and B) and EETs (Fig. 5C-F), as well as the sequential metabolism of EETs to their dihydroxylated metabolites (Fig. 5G-J) at a relatively low concentration of AA (7.81 µM). At a constant AA concentration of 7.81 µM, IR reduced the formation of measured metabolites in the 5-min group. This reduction was significant for 19-HETE, 20-HETE, 5,6-EET, 8,9-EET, 14,15-EET, and 11,12-DHET (Fig. 5), without any significant differences between the Sham and IR groups for the later reperfusion times of 3 or 24 h (Fig. 5). Analysis by ANOVA also revealed a significant effect of reperfusion time on the production rate of metabolites, with the 24-h group generally showing significantly lower rates compared with the 5-min and 3-h groups (Fig. 5). Additionally, in contrast to the *in vivo* concentrations in the liver (Fig. 3), the *in vitro* rates of production of EETs (Fig. 5C-F) in microsomes were much higher than those of their corresponding DHETs (Fig. 5G-J). Further, among DHETs, the rate of 5,6-DHET production (Fig. 5G) was BLQ.

Additional data at the higher substrate (AA) concentration of 62.5 µM are presented in the supplementary material (Fig. S1). The rates of metabolite formation at a higher AA substrate concentration (Fig. S1) showed patterns similar to those at the lower concentration of 7.81 µg/g (Fig. 5). However, the trends towards lower metabolite formation rates in the 5-min IR group did not reach significance at this higher AA concentration (Fig. S1).

### Effects of Hepatic IR Injury on the Liver Microsomal Content of CYP4A, CYP2C23, CYP2J2, and Cytochrome b_5_ and Activities of CPR and mEH

**Fig. 6:**
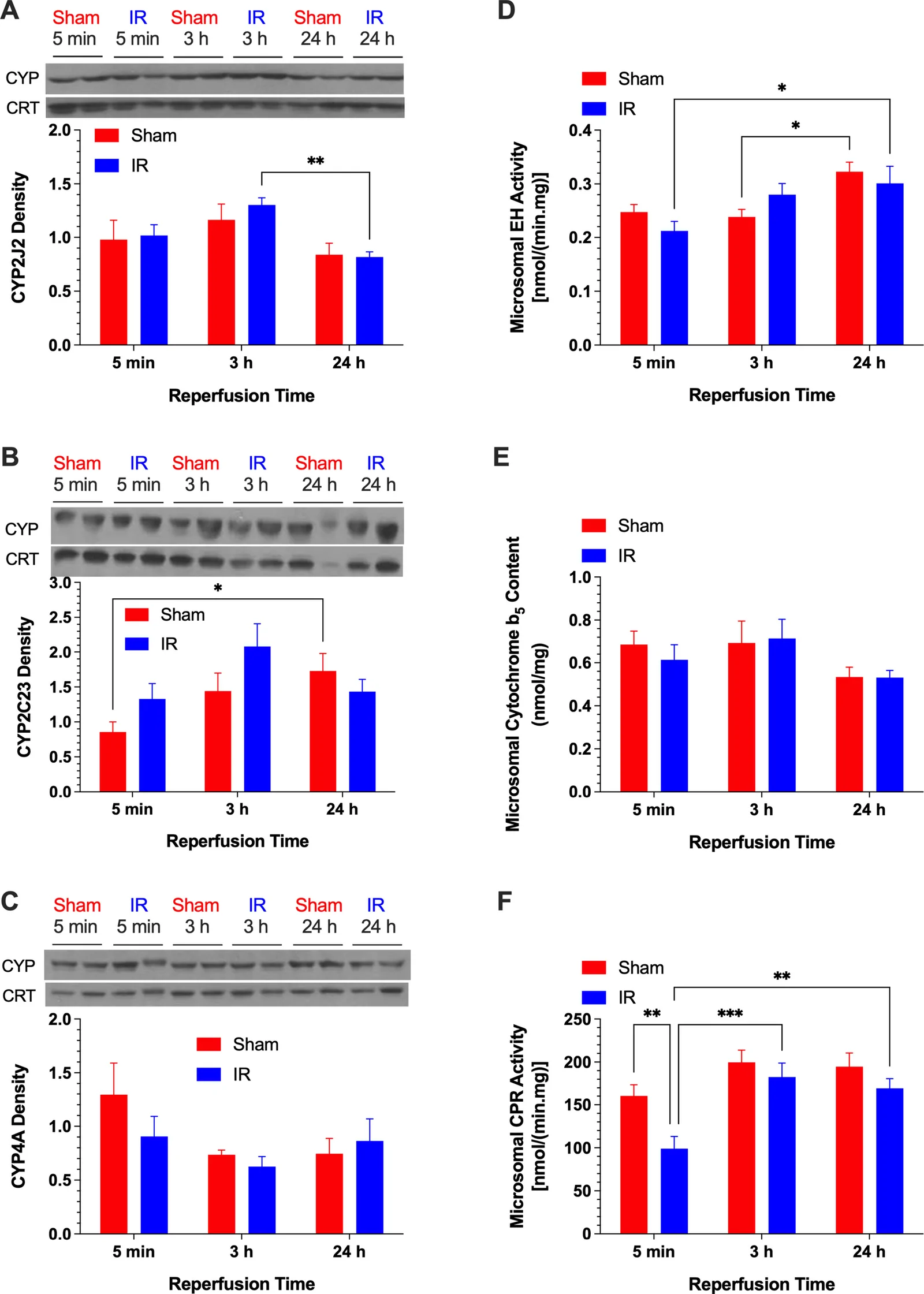
The Western blot analysis of the protein contents of CYP2J2 (**A**), CYP2C23 (**B**), and CYP4A (**C**), along with the epoxide hydrolase (mEH) activity (**D**), cytochrome *b*_*5*_ content (**E**), and CPR activity (**F**) in the liver microsomes of rats subjected to partial hepatic ischemia (IR) or sham operation (Sham) and 5 min, 3 h, and 24 h of reperfusion. Columns and bars represent mean and SE values, respectively. Representative images of the Western blots for each P450 enzyme (CYP) and the loading control calreticulin (CRT) are also presented at the top of panels A-C. The number of animals per group was 6, except for the 3- and 24-h Sham groups, which had 7 and 5 animals, respectively.* *p* < 0.05, ** *p* < 0.01, *** *p* < 0.001: Significant difference between Sham and IR at each time point or between different reperfusion times for Sham or IR, based on two-way ANOVA, followed by Bonferroni’s post-hoc analysis.

Figure 6 depicts the Western blot analysis of the protein contents of CYP2J2 (Fig. 6A), CYP2C23 (Fig. 6B), and CYP4A (Fig. 6C), along with the mEH activity (Fig. 6D), cytochrome *b*_*5*_ content (Fig. 6E), and CPR activity (Fig. 6F) in the liver microsomes of rats subjected to Sham or IR procedure after 5 min, 3 h, or 24 h of reperfusion. IR had no notable impact on P450 enzyme protein levels at any of the time points studied (Fig. 6A-C). Likewise, mEH activities (Fig. 6D) in the IR and Sham rats were comparable across all reperfusion times. Whereas there was no change in the cytochrome *b*_*5*_ content because of IR (Fig. 6E), the microsomal CPR activities in the 5-min IR group were significantly (*p* < 0.01) reduced by 38% compared to the Sham group (Fig. 6F).

## Discussion

The data presented in our current investigation clearly show that the liver concentrations of free AA (Fig. 2) and its major P450 metabolites (Fig. 3) are substantially increased early (5 min) after the reperfusion of the ischemic rat livers. In tissues, AA is primarily stored in lipid membranes at the *sn-*2 position of phospholipids, with only a small fraction being free [25, 44]. It has been reported that various forms of PLA_2_ enzymes, such as sPLA_2_, cPLA_2_, and iPLA_2_, catalyze the hydrolysis of phospholipids at the *sn*-2 position to release AA [8, 44–46]. The significant increases in the activities of various PLA_2_s measured in the liver tissue (Fig. 2B and C) and plasma (Fig. 2D) in the 5-min IR group, relative to the Sham group, are consistent with the significant increases in the concentrations of the unbound AA in this group (Fig. 2A).

Previous studies have shown that warm IR injury causes significant increases in the activity of PLA_2_ enzymes in various organs, including the kidneys [45, 47], brain [46], and heart [48]. Our data on warm liver IR injury (Fig. 2) are consistent with these reports and a study [49] on liver grafts from non-heart-beating human donors subjected to warm IR injury. In contrast, Terao *et al*. [50] reported that hepatic IR injury in rats did not alter the PLA_2_ activity. This apparent discrepancy may be due to differences in the time course of the studies of Terao *et al*. [50] and ours. Whereas we studied PLA_2_ activity early (5 min) during the reperfusion period (Fig. 2), the earliest time point in the study by Terao *et al*. was 1 h after reperfusion [50]. Therefore, the early increase in PLA_2_ enzyme activity observed after warm hepatic IR in our study may have been missed in their studies. Indeed, as in our liver data, others [46] have shown an IR-induced early (10 min after reperfusion) increase in PLA_2_ protein and activity in the brain, which returned to baseline values after 20 min of reperfusion.

Generation of ROS and oxidative stress are among the major hallmarks of hepatic IR injury [4, 6]. It has been reported that excessive oxidative stress causes lipid peroxidation, increasing the activity of PLA_2_ enzymes [44] or enhancing the membranes' susceptibility to their actions [45]. The free concentration of AA in the tissue is a product of the deacylation/reacylation cycle of membrane phospholipids. Oxidative stress can raise unesterified levels of AA not only by promoting hydrolysis (deacylation) of membrane phospholipids and releasing AA but also through a PLA_2_-independent mechanism that reduces its reacylation into phospholipids [44]. Therefore, the substantial increase in free AA concentrations in the liver of the 5-min IR group (Fig. 2A) observed in our studies could be due to both PLA_2_-dependent and independent pathways.

Similar to AA, its P450 metabolites are also incorporated into cellular membrane phospholipids. Karara *et al*. [51] showed the presence of epoxygenated metabolites of AA (EETs), esterified to the glycerol at the *sn*-2 position, in the phospholipid pools of rat livers. In fact, 92% of the endogenous EETs in the rat liver were bound to the membrane phospholipids, with < 1% as free acids. Similar to AA and EETs, P450-generated HETEs, such as 19- and 20-HETE, have also been incorporated into membrane phospholipids, as reported in the kidneys [52], coronary arteries [53], and platelets [54]. Our own previous study [33] demonstrated that a simple freeze–thaw of rat liver resulted in a substantial increase in the free concentrations of all 10 metabolites studied here (Scheme 1), including DHETs. The increases, ranging from 10- (for 14,15-DHET) to 120- (for 5,6-EET) fold, suggest that, similar to AA, the P450 metabolites of AA are highly incorporated into the membrane phospholipids, with only negligible free levels. Therefore, the increased PLA_2_ activity in the 5-min IR group not only releases AA from lipid membranes but is also expected to result in the simultaneous release of preformed P450 metabolites by hydrolysis of phospholipids at the *sn*-2 position.

The pool of free P450 metabolites of AA, measured in our study (Fig. 3), consists of preformed metabolites released from the membrane phospholipids plus de novo production of the metabolites through the metabolism of free AA (Scheme 1). The direct evidence for the metabolism of AA by P450 enzymes was first reported by Capdevila *et al*. [55] using a purified P450 reconstituted system. In both human [56] and rat [42] microsomes, ω/(ω−1) hydroxylated (19- and 20-HETE) and epoxygenated (EETs) metabolites of AA constitute the major portion of the P450-mediated metabolites of AA. To determine if liver IR has any effect on the de novo production of the studied AA metabolites, the rates of P450-mediated metabolism of AA to 19-/20-HETE and EETs and the sequential metabolism of EETs to DHETs by the mEH enzyme were studied in the rat liver microsomes. The IR-induced decreases or no change in microsomal production rates of HETEs, EETs, and DHETs (Fig. 5 and Fig. S1) suggest that changes in the metabolic activities of the P450 enzymes are not responsible for the higher *in vivo* tissue concentrations of the metabolites observed in the 5-min-IR group (Fig. 3).

Whereas the *in vivo* liver concentrations of DHETs were much higher (8,9-DHET, 11,12-DHET, and 14,15-DHET) than or similar (5,6-DHET) to their precursor EETs (Fig. 3), the *in vitro* microsomal rates of production of DHETs were lower than those of their respective EET precursors (Fig. 5). Indeed, the *in vitro* rate of production of 5,6-DHET from 5,6-EET was BLQ (Fig. 5G). The apparent discrepancy between the *in vitro* and *in vivo* data may, at least in part, be due to the fact that in the microsomal preparation, only the microsomal-bound EH (mEH) catalyzes the conversion. However, *in vivo*, in addition to mEH, sEH, which is present in the cytosol [41], also converts EETs to DHETs. Indeed, previous reports [41] have shown that the rate of 14,15-DHET formation from 14,15-EET by the rabbit liver cytosol is 14-fold more than that by the microsomes. Similarly, whereas incubation of synthetic 14,15-EET with rat liver microsomes resulted in only low production of 14,15-DHET, incubation with rat liver cytosol led to a several-fold increase in the formation of the metabolite [57]. The latter study also showed that purified cytosolic EH was substantially more active than its microsomal counterpart. In our studies, we did not assess sEH activity. Therefore, our *in vitro* studies using microsomes have limitations for translation to *in vivo* conditions, as the presence of cytosolic sEH further reduces EET concentrations and increases DHET concentrations *in vivo*.

In addition to investigating the rate of production of the AA metabolites in the microsomes, we also determined the protein contents of the major P450 enzymes responsible for the generation of EETs (CYP2J2 and CYP2C23) and 19-/20-HETE (CYP4A) [25, 43] and the mEH activity for conversion of EETs to DHETs (Fig. 6). IR did not have any significant effects on the protein contents of the measured P450 enzymes or the activity of mEH (Fig. 6). Similarly, IR did not affect the microsomal *b*_*5*_ content, which is known to stimulate the rate of metabolism of AA by P450 enzymes [55]. However, a 38% decrease in CPR activity observed in the 5-min IR group (Fig. 6F) is consistent with comparable reductions (33%–42%) in HETE and EET production rates in microsomes (Fig. 5).

Previous studies have shown that the effects of hepatic IR on P450 proteins and activities are isoenzyme selective [29, 58]. For example, whereas hepatic IR in rats reportedly caused a significant decrease in CYP1A1 content, CYP2E1 protein content increased, and that of CYP2B1 remained unchanged [58]. Further, in a rat model of hepatic IR identical to that used in the current study, we showed that hepatic microsomal CYP2E1 protein content in IR rats after 3 h of reperfusion was not significantly different from that in their Sham counterparts [31]. Therefore, the lack of effect of IR on the protein contents of CYP 4 A, 2C23, and 2J2, observed in our studies, cannot be extrapolated to other potential AA metabolizing P450 isoenzymes like CYP 1A1/2, 2B1/2, or 2C11 (Scheme 1), which were not measured in our studies.

In addition to isoenzyme selectivity, the type of experimental IR model (partial *versus* total ischemia) and variations in the duration of ischemia and reperfusion can greatly influence the results. For instance, different studies have reported reductions [29], no change [59], or increases [60] in cytochrome *b*_*5*_ levels in various experimental models of hepatic IR injury. Therefore, it is important to interpret the findings of different studies within the context of the specific experimental model employed.

Liver reperfusion time significantly affected the liver concentrations of AA (Fig. 2A) and all its measured AA metabolites (Fig. 3), with concentrations in the 24-h groups being significantly lower than those in the earlier time groups. Similarly, the microsomal production rates of most metabolites in the 24-h groups were also lower (Fig. 5). Therefore, both lower substrate availability and lower production rates in the 24-h groups may have contributed to the lower *in vivo* concentrations of AA metabolites in this time group (Fig. 3).

The results of multiple regression analysis indicate that the higher free concentrations of the measured AA metabolites were significantly associated with the higher free concentrations of AA after 5 min of reperfusion, with relatively high *R*^*2*^ values for all metabolites except 14,15-EET (Fig. 4). This association could be due to two processes. First, a higher free AA concentration in the IR group is most likely due to higher PLA_2_ activity (Fig. 2), which hydrolyzes membrane phospholipids at the *sn*-2 position, simultaneously releasing both AA and its preformed metabolites. Therefore, a higher concentration of preformed metabolites is indirectly associated with higher AA levels, as both depend on PLA_2_ activity, which is increased in the IR group. Second, a higher free AA concentration is expected to increase the de novo production of its metabolites directly. However, the increased availability of the substrate (AA) is partially offset by a decrease in the rate of P450 enzymatic activity in the 5-min IR group (Fig. 5). Nevertheless, our study did not distinguish between preformed and de novo metabolites. Therefore, it is not possible to determine the contributions of preformed metabolite release and de novo metabolite synthesis to the increased liver metabolite concentrations observed in the 5-min IR group (Fig. 3).

Although the effects of P450-mediated metabolites of AA on the pathophysiology of liver IR injury have not been studied, recent studies have reported that 12- and 15-HETE metabolites of AA generated by arachidonate 12/15-lipoxygenase (ALOX12/15) play a detrimental role in this injury [22–24]. In 2018, Zhang *et al*. [22] reported that ALOX12 was substantially upregulated in hepatocytes during ischemia. The upregulation of ALOX12 led to increased production and accumulation of 12-HETE, triggering a cascade of inflammatory processes in mice. The authors also showed that the inhibition of 12-HETE production via ALOX12 inhibitors prevented hepatic IR injury in mice, pigs, and nonhuman primates. This study suggested that ALOX12 inhibitors could be an effective strategy for mitigating hepatic IR injury. Recent studies have confirmed that, whereas the activation of the ALOX15/15-HETE pathway is detrimental [23], inhibition of 15-HETE production is protective in mouse models of hepatic IR. Although mid-chain HETEs such as 5-, 11-, 12-, and 15-HETE are primarily formed by lipoxygenases, they can also be generated by some P450 isoenzymes, such as CYP1B1 and CYP1A1, which exhibit lipoxygenase-like activity [61]. Our study is limited in that we did not measure lipoxygenase levels or those of CYP1B1 or CYP1A1.

It has been reported that the liver P450 enzymes responsible for AA metabolism are sex-dependent in rats [62]. For example, whereas the protein contents of CYP1A2 and CYP4A1 were higher in female rats, the CYP2J2 protein was higher in males. Additionally, CYP2C11 and CYP4A2 are male-specific in the rat liver. In the studies presented here, we used only male rats. However, it is possible that the generation of de novo metabolites of AA may follow a different pattern in female rats.

## Conclusions

Warm (normothermic) hepatic IR in rats caused an increase in the liver concentrations of free (unesterified) AA and its major P450-mediated hydroxylated and epoxygenated metabolites early after reperfusion. The IR-induced early increase in liver concentrations of the free AA and its metabolites was associated with a concomitant increase in PLA_2_ enzyme activity. In contrast, hepatic IR significantly reduced or did not change the *in vitro* metabolic activities of AA-metabolizing P450 enzymes. The IR-induced increase in PLA_2_ activity suggests that the observed increase in metabolite concentrations in the IR livers is due, at least in part, to the increased release of the preformed metabolites from lipid membranes. However, the contribution of the de novo formation of the metabolites due to an increased substrate (AA) availability, despite a reduction in the CPR-mediated P450 enzymatic activity, cannot be ruled out. Further studies are needed to determine the exact contributions of each pathway (increased release from membranes and de novo production) to the observed increase in free levels of P450-mediated AA metabolites and their roles in hepatic IR injury.

## Supplementary Information

Below is the link to the electronic supplementary material.

ESM 1 (The in vitro rates of metabolism of AA to its P450-mediated metabolites at an AA concentration of 62.5 µM are presented in the supplementary file. DOCX 1.11 MB)

## Data Availability

The data used in preparation of figures may be obtained from the corresponding author upon reasonable request.
