# Supplementary material for: Effects of Normothermic Hepatic Ischemia–Reperfusion Injury on the Hepatic Disposition of Arachidonic Acid and Its Cytochrome P450-Generated Metabolites in Rats
Source: Pharm Res. 2026 May 23;43(8):2667–82. doi: 10.1007/s11095-026-04108-1 (PMC13593798; doi:10.1007/s11095-026-04108-1)
Supplement: Supplementary file 1 [file 11095_2026_4108_MOESM1_ESM.docx]

**Supplementary Material**

**Effects of Normothermic Hepatic Ischemia-Reperfusion Injury on the Hepatic Disposition of Arachidonic Acid and Its Cytochrome P450-Generated Metabolites in Rats**

Vindhya Edpuganti . Reza Mehvar

**Fig. S1** The in vitro rate of metabolism of AA (62.5 µM) to HETEs (A and B) and EETs (C-F) and sequential metabolism of EETs to their dihydroxylated (DHETs) metabolites (G-J) in the liver microsomes of rats subjected to partial hepatic ischemia (IR) or sham operation (Sham) and 5 min, 3 h, and 24 h of reperfusion. Columns and bars represent mean and SE values, respectively. The number of animals per group was 6, except for the 3- and 24-h Sham groups, which had 7 and 5 animals, respectively.* *p* < 0.05, ** *p* < 0.01, *** *p* < 0.001, **** *p* < 0.0001: Significant difference between Sham and IR at each time point or between different reperfusion times for Sham or IR, based on two-way ANOVA, followed by Bonferroni’s post-hoc analysis. BLQ, below the limit of quantitation of the assay.
